# Presence of Senescent and Memory CD8+ Leukocytes as Immunocenescence Markers in Skin Lesions of Elderly Leprosy Patients

**DOI:** 10.3389/fimmu.2021.647385

**Published:** 2021-03-11

**Authors:** Pedro Henrique Lopes da Silva, Katherine Kelda Gomes de Castro, Mayara Abud Mendes, Thyago Leal-Calvo, Júlia Monteiro Pereira Leal, José Augusto da Costa Nery, Euzenir Nunes Sarno, Roberto Alves Lourenço, Milton Ozório Moraes, Flávio Alves Lara, Danuza Esquenazi

**Affiliations:** ^1^Laboratório de Hanseníase, Instituto Oswaldo Cruz, Fundação Oswaldo Cruz, Rio de Janeiro, Brazil; ^2^Laboratorio de Envelhecimento Humano, GeronLab, Policlínica Piquet Carneiro, Universidade do Estado do Rio de Janeiro, Rio de Janeiro, Brazil; ^3^Laboratório de Microbiologia Celular, Instituto Oswaldo Cruz, Fundação Oswaldo Cruz, Rio de Janeiro, Brazil; ^4^Disciplina de Patologia Geral, Faculdade de Ciências Médicas, Universidade do Estado do Rio de Janeiro, Rio de Janeiro, Brazil

**Keywords:** leprosy, elderly, immunosenescence, skin lesions, memory T cell, cytomegalovirus

## Abstract

Leprosy is an infectious disease that remains endemic in approximately 100 developing countries, where about 200,000 new cases are diagnosed each year. Moreover, multibacillary leprosy, the most contagious form of the disease, has been detected at continuously higher rates among Brazilian elderly people. Due to the so-called immunosenescence, characterized by several alterations in the quality of the immune response during aging, this group is more susceptible to infectious diseases. In view of such data, the purpose of our work was to investigate if age-related alterations in the immune response could influence the pathogenesis of leprosy. As such, we studied 87 individuals, 62 newly diagnosed and untreated leprosy patients distributed according to the age range and to the clinical forms of the disease and 25 healthy volunteers, who were studied as controls. The frequency of senescent and memory CD8^+^ leukocytes was assessed by immunofluorescence of biopsies from cutaneous lesions, while the serum levels of IgG anti-CMV antibodies were analyzed by chemiluminescence and the gene expression of T cell receptors' inhibitors by RT-qPCR. We noted an accumulation of memory CD8^+^ T lymphocytes, as well as reduced CD8^+^CD28^+^ cell expression in skin lesions from elderly patients, when compared to younger people. Alterations in *LAG3* and *PDCD1* gene expression in cutaneous lesions of young MB patients were also observed, when compared to elderly patients. Such data suggest that the age-related alterations of T lymphocyte subsets can facilitate the onset of leprosy in elderly patients, not to mention other chronic inflammatory diseases.

## Introduction

Leprosy is a neglected chronic infectious disease caused by *Mycobacterium leprae*, which remains a public health problem in low-income countries (1, 2). *M. leprae* affects mainly the skin and peripheral nervous system, where the bacilli are responsible for neurological damage, bone resorption, and irreversible physical disabilities (3–5). Genetic and environmental factors contribute to disease progression (3). Fortunately, around 95% of people are genetically resistant to *M. leprae* infection (3, 6).

Leprosy presents a wide spectrum of clinical forms, which is essentially determined by the presence (or absence) of cell-mediated immunity (CMI) against the pathogen. According to the Ridley and Jopling classification (7), tuberculoid forms (T-Lep) are characterized by a strong immune response to localized disease with a single (or few) skin lesion(s) without bacilli detection. On the other side, lepromatous forms (L-Lep) are characterized by several disseminated skin lesions with many bacilli, and the absence of CMI against *M. leprae*. Between these polar forms (TT and LL) there are borderline forms (BT, BB, and BL), which comprise most of the patients. In addition, there is also a WHO classification, a more practical and operational classification, which classifies leprosy patients into two groups: paucibacillary leprosy (PB leprosy) that presents five or less skin lesions and no apparent bacilli in slit-skin smears; and multibacillary leprosy (MB leprosy) with more than five skin lesions (8).

During the aging process, significant changes in the composition of peripheral T lymphocytes occur (9). These changes are mostly caused by decline in the output of naïve T cells due to thymic involution and exposure to pathogens over the lifetime, especially latent viruses, such as cytomegalovirus (CMV), which are related to the accumulation of highly differentiated CD8^+^ memory T cells (9, 10). Although a mechanism of compensation by homeostatic proliferation partly maintains the numbers of naïve T cells in the periphery, the adaptive immune system in elderly people is characterized by higher proportions of memory T cells and lower proportions of naïve T cells (11). CD8^+^ T cells play a central role in the recognition and clearance of intracellular pathogens, such as *M. leprae*. CD8^+^ T cell frequency is similar between the leprosy clinical forms, although functional features such as higher IL-10 levels in MB compared to PB patients in this T cell subset has been observed (12). Moreover, TNF-producing CD8^+^ T cells are essential in the pathogenesis of the type 2 leprosy reaction in MB patients, these reactions are episodes of acute hypersensitivity presenting as aggravation of the previous symptoms and skin lesions (13).

During the aging process, the T cell costimulatory molecule, CD28 (needed for activation and survival), is lost, interfering in cellular signaling and functional aspects of these cells (14, 15). The binding of CD28, expressed on the T cell surface, to CD80 or CD86 stimulates IL-2 production leading to T cell activation and proliferation, while signals through the TCR/CD3 complex in the absence of a costimulatory signal induces anergy (16). Recent works have shown that the loss of CD28 expression is a hallmark of senescent CD8^+^ T cells, and proportion changes in CD8^+^CD28^−^ cells have been reported in aging-related diseases such as cancer, cardiovascular disease, and other chronic inflammatory diseases (17–19). Accumulating evidence indicates that CD8^+^CD28^−^ T cells play a relevant role in immune response suppression, such as impairing T cell activation and proliferation, and inducing apoptosis in these cells *in vitro* (20, 21). The immunosuppressive mechanisms performed by these cells are diverse, including secretion of anti-inflammatory cytokines, increased expression of programmed cell death protein 1 (PD-1) and its ligands, and FasL (22, 23). Several studies showed that the CD28 constitutive expression level is similar between PB patients and healthy individuals, while MB patients presented lower CD28 expression (24–26). This suggests that upregulation of the CD28 molecule plays a critical role in the cell-mediated immunity response against *M. leprae*, restricting the proliferation of the pathogen.

The present study aimed at investigating potential age-related alterations that are associated with CD8^+^ cells in elderly leprosy patients. For this purpose, blood and skin lesion samples from young and elderly patients were evaluated for the frequency of memory and senescent CD8^+^ T cells, anti-CMV IgG titers, and gene expression of inhibitory T cell receptors. We observed the accumulation of memory CD8^+^ cells in the skin lesions of all elderly patients, followed by a lower frequency of CD8^+^CD28^+^ in elderly PB patients compared to young ones. We also observed changes in gene expression of the *LAG3* and *PDCD1* receptors in cutaneous lesions of young MB patients and not in elderly leprosy patients.

## Methods

### Participants and Study Design

**Figure 1:**
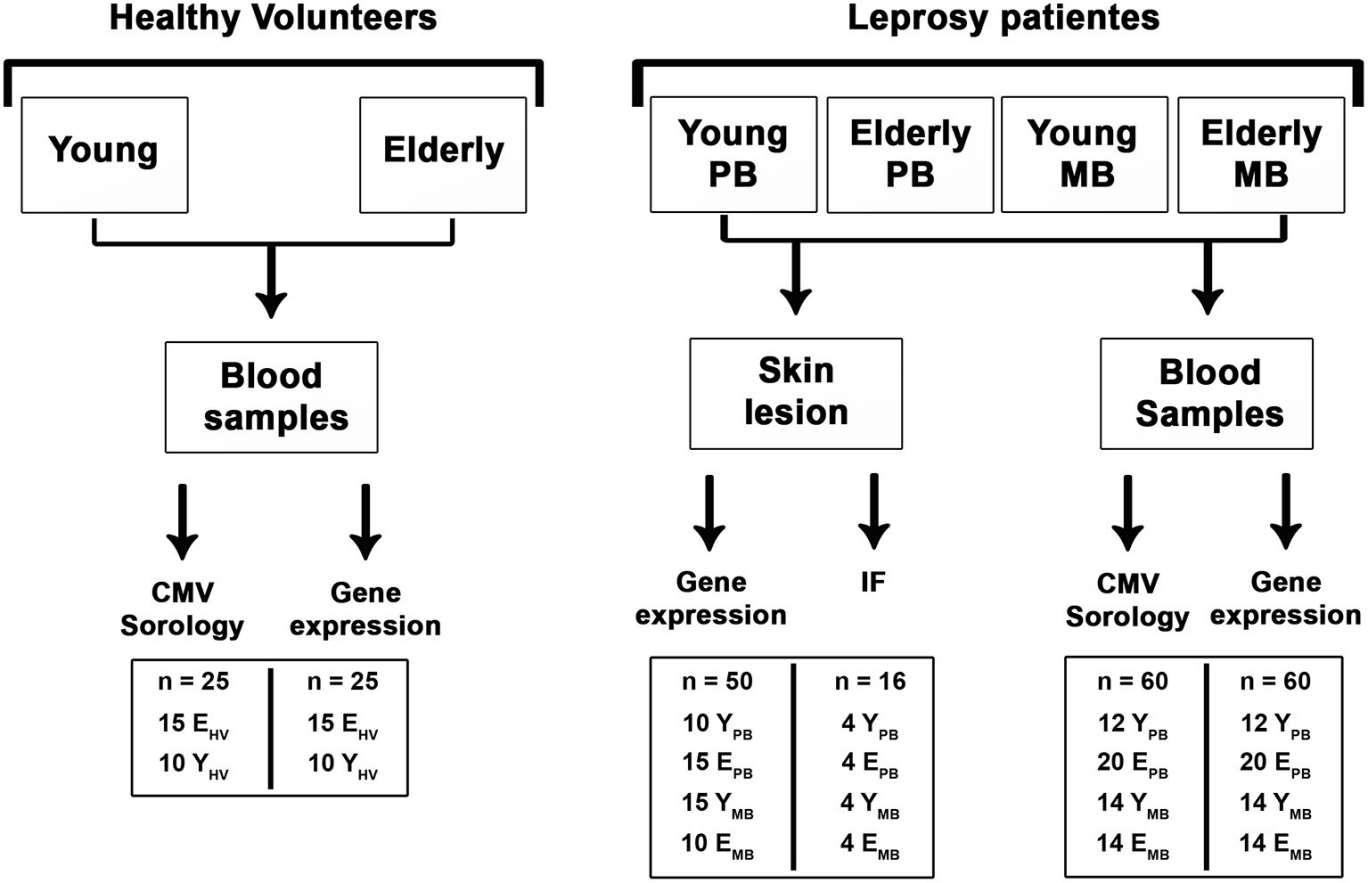
Study design. Groups of individuals evaluated and tests performed. A total of 62 leprosy patients and 25 healthy volunteers were clinically and/or laboratory assessed. At each analysis performed, the numbers of individuals tested by group are shown (inside the boxes).

All enrolled leprosy patients were classified according to the Ridley and Jopling scale (1966), then the diagnosis was confirmed by clinical examination and histopathological analysis of skin lesions. Blood and skin lesion samples were collected before treatment (Figure 1). Patients were classified according to clinical forms and also stratified into two groups: young (20–40 years old) and elderly (over 60 years old). All patients and healthy volunteers resided in the metropolitan region of Rio de Janeiro state, Brazil, a leprosy endemic area. Exclusion criteria for leprosy patients and healthy elderly volunteers were: relapse cases, pregnancy or breast-feeding women, co-infections such as tuberculosis, hepatitis B and C, and HIV. Hypertensive and diabetic elderly individuals under drug control were included. As previously mentioned, our work used clinical samples of young and elderly patients diagnosed according to the R&J criteria. Nevertheless, in order to avoid repetitions, BL and LL individuals shall be hereinafter grouped as MB patients, and BT and TT as PB patients.

### Ethical Considerations

The study was approved by the Institutional Ethics Committee of Oswaldo Cruz Foundation/FIOCRUZ (permit protocol number 27052919.0.0000.5248). Leprosy patients and healthy volunteers signed a written consent form to participate in the study. The biological samples from leprosy patients were obtained at the Leprosy outpatient clinic (FIOCRUZ/Rio de Janeiro). Elderly healthy individuals were recruited at the Human Aging Laboratory – GeronLab, Policlínica Piquet Carneiro (UERJ/Rio de Janeiro).

### Immunofluorescence Assay

Frozen skin lesion section assays were performed in a Leica LM3000 cryostat, fixed in paraformaldehyde. Unspecific binding sites were blocked with 10% Fetal Calf Serum (FCS, GIBCO, Life Technologies) in 0.01 M of PBS for 1 h at room temperature. Permeabilization was performed by incubating the sections with 0.05% Triton X-100 for 15 min. Rat IgG2b anti-human CD8 (1:50; Abcam, ab60076), mouse IgG2a anti-human CD45RO (1:25; Abcam, ab86080), and rabbit IgG anti-human CD28 (1:50; Abcam, ab243228) or their respective isotypes were diluted in 1% Bovine Serum Albumin (BSA, Sigma-Aldrich) in 0.01 M of PBS and incubated at 4°C overnight. Tissue sections were washed three times and incubated with Alexa Fluor 594 goat anti-Rat IgG (1:1,000, Abcam, ab150164), Alexa Fluor 633 goat anti-mouse IgG1 (1:1,000, ThermoFisher Scientific, A-21126), and Alexa Fluor 488 goat anti-rabbit IgG (1:1,000, Abcam, ab150077) secondary antibodies for 1.5 h at room temperature. The nuclei were stained with 4′-6-diamidino-2-phenylindole (DAPI; 1:10,000, Molecular Probes, D1306), and slides were mounted with VECTASHIELD Mounting Medium (Vector Laboratories, H-1000). Tissues were imaged using an Axio Observer.Z1 (Carl Zeiss, Oberkochen, Germany) using an EC Plan-Neofluar 20×/0.50 objective and Plan-Apochromat 63×/1.3 oil objective. Images were acquired with an AxioCam HRm digital camera, in the format of structured confocal images by Apotome (Carl Zeiss), mathematically deconvoluted by the AxioVision Rel. 4.6 software (Carl Zeiss). For quantitative analysis of CD8^+^, CD45RO^+^, and CD28^+^ cells, 10 microscopic fields were imaged and the number of positive cells was counted in each field. The results were summarized as the average of fields' counts, as determined by three independent observers.

### CMV Serology

IgG antibodies against CMV were determined in sera samples with a commercially available chemiluminescent microparticle immunoassay according to the manufacturer's instructions (Architect CMV IgG, Abbott Laboratories, Diagnostic Division, Sligo, Ireland). Absorbance was measured and an optical density (OD) ratio was calculated. The default result unit for the assay was AU/mL. Specimens with concentration values ≥15.0 AU/mL were considered reactive for IgG antibodies to CMV and indicated past or acute infection.

### Total RNA Isolation and cDNA Synthesis

The total RNA from whole blood obtained by venous puncture was isolated using the PAXgene^TM^ Blood RNA kit (Qiagen, Hilden, Germany) in accordance with the manufacturer's instructions. For biopsy specimens, RNA from skin lesion samples (6 mm^3^ punch) was obtained using the Polytron Homogenizer Model PT3100 apparatus (Kinematica AG, Lucerne, Switzerland) in 2 mL of TRIzol™ Reagent (ThermoFisher Scientific, Massachusetts, USA), following the manufacturer's instructions. Total RNA was treated with TURBO™ DNase (ThermoFisher Scientific) according to the manufacturer's standard protocol, quantified, and their integrity was evaluated by agarose gel electrophoresis. RNA reverse transcription into cDNA was performed as previously described (27).

### Gene Expression Analysis by RT-qPCR

Quantitative RT-PCR was carried out with a final volume of 10 μL containing 200 nM of each primers (Supplementary Table 1), 1X Fast SYBR^TM^ Green Master Mix (ThermoFisher Scientific), and 10 ng of cDNA. All reactions were conducted with three technical replicates for each biological sample. No reverse transcriptase negative controls and no template controls were included in each run. The assays were performed on a StepOnePlus^TM^ Real-Time PCR Systems thermocycler (ThermoFisher Scientific) as detailed elsewhere (27). The relative expression levels of the genes of interest were normalized by ribosomal protein L13. qPCR data analysis was performed with the N_0_ method implemented in LinRegPCR v. 2020.0, which considers qPCR mean efficiencies estimated by the window-of-linearity method as proposed by Ramakers et al. (28) and Ruijter et al. (29). Briefly, N0 values were calculated in LinRegPCR using default parameters. Then, N0 values from the gene of interest (GOI) were normalized by taking its ratio to the N0 of the reference gene (REF) *RPL13a* (N0_GOI_/N0_REF_).

### Biomark Fluidigm Gene Expression

Gene expression from whole blood was measured using Biomark's microfluidic-based qPCR technology. cDNA was obtained from RNA as described above. Then, 1.25 μL of cDNA (from stock concentration of 5 ng/μL) was pre-amplified with a pool of 96 primer pairs (final concentration of 50 nM) with the TaqMan PreAmp Master Mix 2X (Applied Biosystems, USA, # 4391128) in a GeneAmp PCR System 9700 thermocycler for 14 cycles. All subsequent steps are detailed in research by Guerreiro et al. (27). For data analysis, initial quality control was performed based on melting curve analysis (MCA) using the Fluidigm Real-Time PCR Analysis Software v. 4.5.2, where targets with multiple dissociation curve peaks were removed from further analysis. Later, raw data were exported and processed with custom R scripts as described elsewhere by Guerreiro et al. (27). In brief, foreground data (EvaGreen) were adjusted by the subtraction of background (Rox) intensity to generate Rn (background-adjusted accumulated fluorescence). Then, qPCR reaction efficiency was estimated by fitting a four-parameter sigmoid model according to Rutledge and Stewart (30), with functions from the R package qpcR v.1.41-1 (31). Cycle thresholds (Ct) were determined from the maximum of the second derivative from the fitted sigmoid curve. Cts and efficiencies were used to estimate relative expression based on the method proposed by Pfaffl (32). The normalization factor used in the denominator for relative expression consisted of the geometric mean from *RPS16, RPL13*, and *RPL35* genes, selected as the most stable by the R implementation of the geNorm algorithm (33, 34).

### Statistical Analysis

Results were analyzed using Statistical Package for the Social Sciences (SPSS) V. 10.1 (SPSS, Inc., Chicago, IL, USA) and Graph Prism V. 8 (San Diego, CA, USA) software. After testing for normality (Shapiro-Wilk normality test), non-normally distributed data were analyzed through non-parametric tests. The Mann/Whitney U-test was used to test the differences between two groups, and comparisons between more than two groups were examined through the Kruskal-Wallis test followed by *post-hoc* Dunn's correction. Normally distributed data were compared using one-way ANOVA followed by Tukey's multiple comparisons test. A *P*-value < 0.05 was considered statistically significant.

## Results

### Subject Demographics and Clinical Data

The present study enrolled 87 individuals, 62 of whom were patients diagnosed with leprosy and 25 were healthy volunteers (HV). Both patients and HV were divided into two groups according to age: young (20–40 years age) and elderly (60 years age or older). The mean age of the young groups was 30 years of age (29.3 for young HV, 29.3 for young PB, 31.7 for young MB). In the elderly groups, the mean ages were 79.1, 69.1, and 67.7 years for elderly HV, elderly PB, and elderly MB patients, respectively. Participants' clinical and demographic data are shown in Table 1.

**Table 1:** Characteristics of study population.

**Groups**	***N***	**Age, years (Mean ± SD)**	**Sex (%male)**	**BI (Mean ± SD)**	**LBI (Mean ± SD)**	**DG (%)**			**Education level (Mean ± SD)**	**Time to diagnosis (median)**
						**0**	**I**	**II**		
Y HV	10	29.3 ± 7.1	40	–	–	–	–	–	16.1 ± 5.7	–
E HV	15	79.1 ± 6.3	46.7	–	–	–	–	–	10.4 ± 4.1	–
Y PB	12	29.3 ± 6.2	66.7	0	0	83.3	16.7	0	10.1 ± 4.3	12
E PB	20	69.1 ± 7.5	30	0	0	90	5	5	5.9 ± 4.4	10
Y MB	15	31.7 ± 3.2	60	4.5 ± 1.2	4.9 ± 1.1	53.3	33.3	13.4	7.9 ± 4.3	11.5
E MB	15	67.7 ± 6.7	73.3	4.1 ± 1.1	3.7 ± 1.4	33.3	46.7	20	3.5 ± 2.8	11.5

*Y, Young; E, Elderly; HV, Healthy Volunteers; PB, Paucibacillary leprosy; MB, Multibacillary leprosy; BI, Bacillary Index; LBI, Lesion Bacillary Index; DG, Disability Degree*.

According to the Ridley and Jopling classification of patient clinical spectrums, those in the MB group were classified as BL (40%) or LL (60%), while PB patients were either TT (12.5%) or BT (87.5%). Among elderly MB patients, there was a high frequency of male individuals when compared to the other groups. This unequal gender distribution was expected due to the influence of sex hormones on the immune response to infectious diseases. Lower education level (average of 5 years) of elderly patients was not related to a delay in diagnosing the disease. Although young patients had higher education levels when compared to elderly patients (*p* = 0.0035), especially in the PB group (10 years), the time from symptoms onset to disease diagnosis was, on average, 1 year for both young and elderly participants.

Bacterial load, as measured by the bacterial index (BI) in slit skin smears samples, was greater in young MB patients than in the elderly patients, although this difference was not significant (*p* = 0.2932). On the other hand, bacterial lesion index (BLI) was significantly greater in young MB patients than in elderly patients (*p* = 0.0112). This information is particularly interesting considering that the frequency of MB patients in young and elderly groups was similar.

### Memory Cutaneous CD8^+^ Cell Populations are More Frequent in Elderly Leprosy Patients

**Figure 2:**
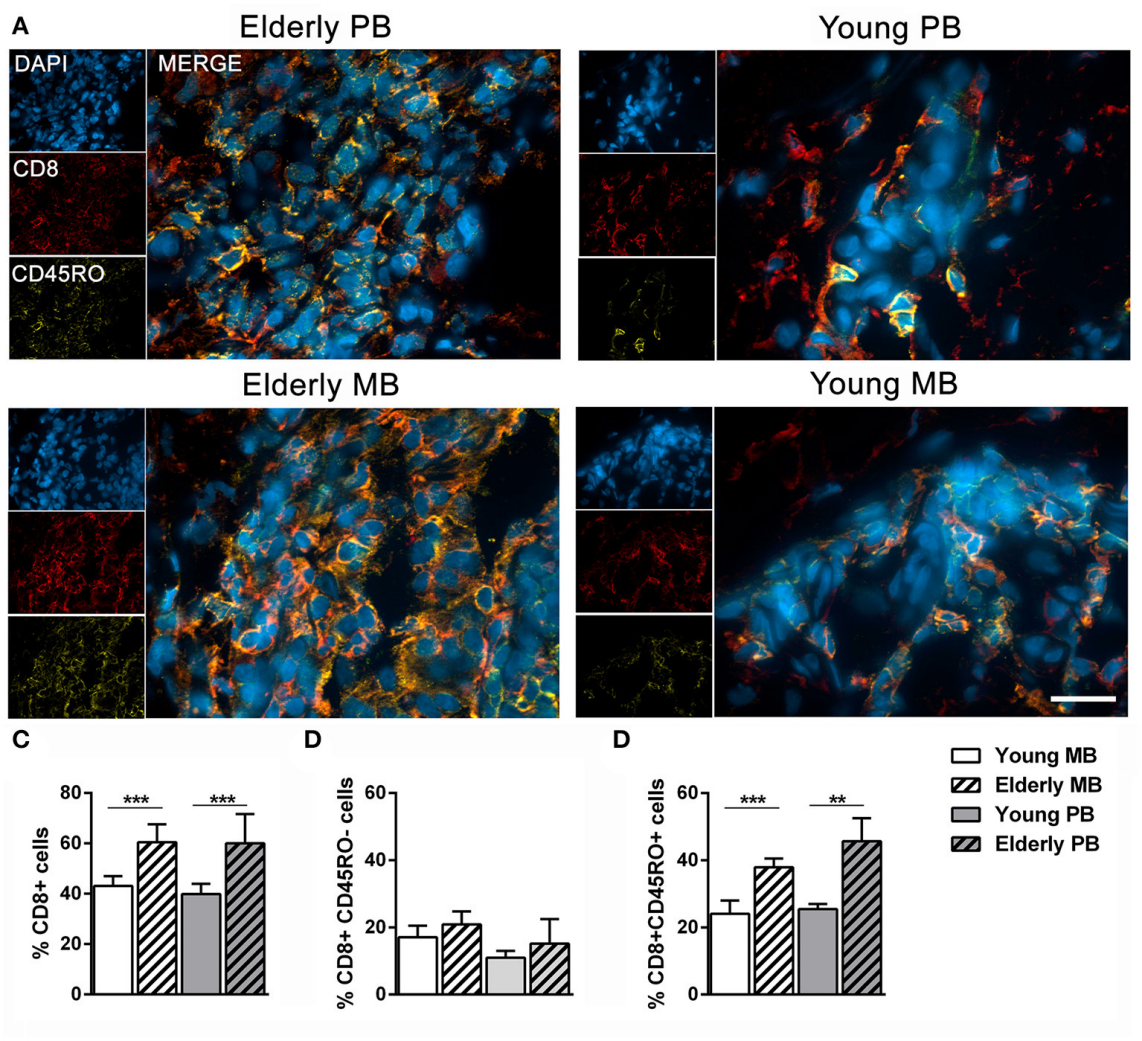
Frequency of memory CD8^+^ T cells in skin lesions samples. Immunofluorescence assays were performed to determine the number of memory CD8^+^ T cells (CD8^+^CD45RO^+^) in skin lesions of leprosy patients. The images are representative of a patient from each group of four individuals, where CD8 (red, Alexa Fluor 594), CD45RO (yellow, Alexa Fluor 633), and the nuclei (blue) were stained with DAPI **(A)**. The images were visualized and obtained by a Zeiss Colibri fluorescent microscope, and the scale bar was 20 μm. The graphs show the frequency of **(B)** CD8^+^ T cells, **(C)** CD8^+^CD45RO^−^ T cells, and **(D)** CD8^+^CD45RO^+^ T cells. Analysis was performed by one-way ANOVA followed by Tukey's multiple comparison test. Data are representative of four individuals in each group and the results are shown as means ± SD, where **P < 0.01, and ***P < 0.001.

Memory CD8^+^ cells (CD8^+^CD45RO^+^) presence was detected in patients' skin lesions by labeling with anti-CD8 and anti-CD45RO antibodies, as described in the methods section. Figure 2A represents results from the four studied groups. CD8^+^ leukocytes were significantly higher in elderly skin lesions when compared to those of young patients, regardless of clinical form (Figure 2B). Memory CD8^+^ cells (CD8^+^CD45RO^+^) were also greater in elderly than in young patients (Figure 2C), irrespective of clinical form. On the other hand, naïve cells (CD8^+^CD45RO^−^) were not significantly different in skin lesions from young and elderly patients (Figure 2D). As expected, these results demonstrate an accumulation of memory CD8^+^ cells in elderly patient skin lesions, which is a process linked to immunosenescence.

### Low Frequency of CD8^+^CD28^+^ Cells in Skin Lesion Samples of Elderly Leprosy Patients

**Figure 3:**
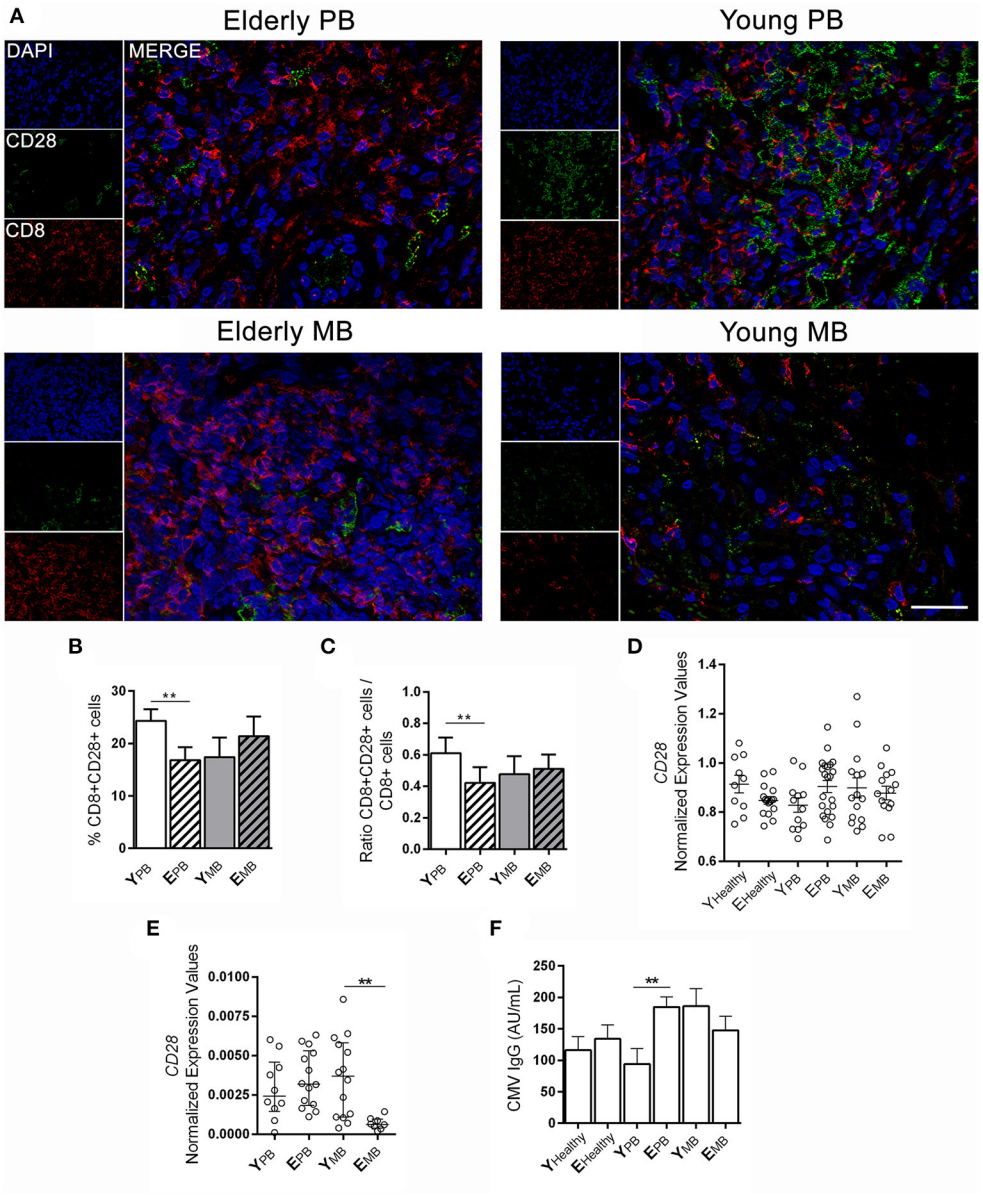
CD28 expression in blood and skin lesion samples. Fluorescence immunostaining of CD28 (green) and CD8 (red) in skin lesions. **(A)** The images are representative of a patient from each group of four individuals. Images were visualized and obtained by a Zeiss Colibri fluorescent microscope and the scale bar was 20 μm. The graphs show the frequency of **(B)** CD8^+^CD28^+^ T cells, **(C)** ratio between CD8^+^CD28^+^ cells and CD8^+^ T cells, and the data are demonstrated by means ± SD. The results of CD28 gene expression in **(D)** blood samples and **(E)** skin lesion samples are shown in normalized expression values. Whole blood samples were measured using Biomark's microfluidic-based qPCR technology and experiments with skin lesions were performed with RT-qPCR. Each circle represents an individual, horizontal bars indicate the mean. **(F)** IgG anti-CMV antibodies in sera samples in the studied groups were performed by a chemiluminescence method. Bars represent mean ± SD. Analysis was performed by one-way ANOVA followed by Tukey's multiple comparison test, and **P < 0.01.

Although CD8^+^ T lymphocytes play an important part in controlling *M. leprae* proliferation, the presence of these senescent lymphocytes (CD8^+^CD28^−^) may hinder the control of infection due to their regulatory T cell activities. Figure 3A presents CD8 and CD28 expression in skin lesions of the groups of patients in this study. CD8^+^CD28^+^ cells were significantly more frequent in young PB than elderly PB patients in skin lesions (*p* = 0.0015; Figure 3B). However, the same was not observed among patients in MB groups, in which both young and elderly exhibited similar levels of CD8^+^CD28^+^ cells (Figure 3B). Ratios were calculated between CD8^+^CD28^+^ and total CD8^+^ cells in order to confirm the higher frequency of CD8^+^CD28^+^ cells in young PB patients when compared to total CD8^+^ cells (Figure 3C).

Gene expression of co-stimulating *CD28* in whole blood samples showed no difference in *CD28* mRNA expression in blood across the studied groups (Figure 3D). However, when it comes to *CD28* mRNA expression in skin lesions by RT-qPCR, elderly MB patients showed a significant reduction when compared to other groups in the study, including young patients with the same clinical form (Figure 3E; *p* = 0.0024).

Latent subclinical infection caused by CMV may lead to immunological alterations linked to aging, such as the accumulation of memory CD8^+^ cells that do not express CD28. We carried out detection of anti-CMV IgG in the serum of studied participants. All elderly patients were positive for anti-CMV IgG, while 85% of young patients were positive. The lowest frequency of anti-CMV IgG positive individuals occurred in the young and healthy group (72%). However, antibody titers were not correlated to CD8^+^CD28^+^ cells in any of the studied groups. Furthermore, in elderly PB patients, only those with significantly lower CD8^+^CD28^+^ cells presented higher anti-CMV IgG levels, as opposed to young PB patients (Figure 3F; *p* = 0.0054).

### Regardless of Age, MB Patients Presented Higher Gene Expression of T Cell Inhibitory Receptors Than PB Patients

Regulatory T lymphocytes use diverse strategies to suppress the immune response mediated by other subpopulations of T lymphocytes. Therefore, we hypothesized that PD-1 and LAG3 T lymphocyte inhibition receptors (also known as CD279 and CD223, respectively) could be part of the suppression strategy, linked to greater susceptibility to infectious diseases such as leprosy. Furthermore, CMV infection may induce these inhibition receptors.

**Figure 4:**
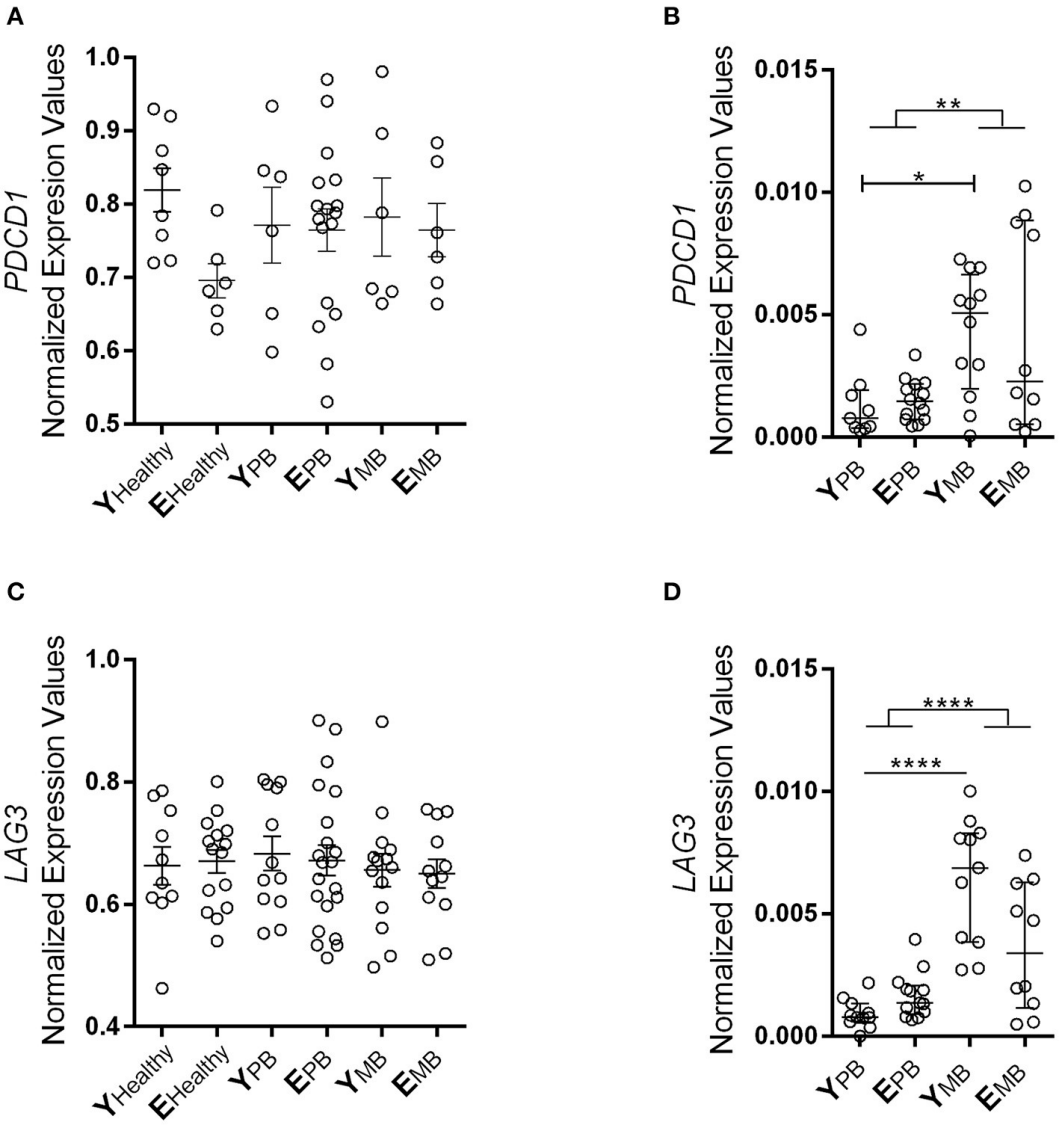
PD-1 and LAG3 gene expression in whole blood and skin lesion samples. Expression of *PDCD1* gene on **(A)** blood and **(B)** skin samples. Expression of *LAG3* gene on **(C)** blood and **(D)** skin lesion samples. The results of these two inhibitory receptors are represented in normalized expression values. Whole blood samples were measured using Biomark's microfluidic-based qPCR technology and fragments of lesions were performed by RT-qPCR. Each circle represents an individual, and the horizontal bars indicate the mean. Kruskal-Wallis test with Dunn's multiple comparison test correction was used to compare the groups. *P < 0.05, **P < 0.01, and ****P < 0.0001.

We carried out gene expression analysis of programmed cell death 1 (*PDCD1*) receptor and its ligands *PDCD1lg1* and *PDCD1lg2*, as well as lymphocyte activation protein (*LAG3*) in blood and skin lesion samples. Blood samples from patients and healthy controls showed similar expression levels of *LAG3* and *PDCD1* (Figures 4A,B). In skin lesions, differences were observed between groups regardless of age. MB patients presented increased expression of *LAG3* and *PDCD1* in skin lesions in comparison to PB patients (*p* < 0.0001, *p* = 0.0031, respectively; Figures 4C,D). Furthermore, young MB patients presented significantly higher expression in skin lesions than PB patients (*p* = 0.0219).

It seems that *LAG3* and *PDCD1* could be associated with mechanisms leading to weaker T lymphocyte response to *M. leprae* in MB patients. However, the expression of these receptors does not seem to be related to aging, at least not under these studied conditions. Then, we analyzed putative age-related changes in programmed cell death receptor (*PDCD1*, i.e., *PDCD1lg1* and *PDCD1lg2*) gene expression in blood and skin lesions (Supplementary Figure 1) and no differences were observed among all groups tested. *PDCD1lg1* and *PDCD1lg2* also demonstrated no difference in gene expression between PB and MB patients, as opposed to *PDCD1*.

## Discussion

The aging process leads to a myriad of innate and adaptive immune system adaptations, which result in increased susceptibility to infections, reduction in vaccine response, and even greater incidence of cancer (35). Although these alterations are related to T cell mediated immune response, the mechanisms by which they increase susceptibility to infectious diseases remain unknown (36). Cunha and collaborators addressed these factors among elderly people affected by SARS-CoV-2 infection (37). Considering that *M. leprae* is capable of infecting a large amount of people, and only a minority will progress toward the disease (1), factors behind delayed disease onset that could be associated with changes in T lymphocyte subpopulations remain unclear. Studies in this area are even more relevant considering epidemiological tendencies of increased disease detection in the population over 60 years of age and stabilization in those between 15 and 60 years (38, 39), which could be increased in a transition from high-endemic to middle-endemicity.

The social impact of leprosy is high due to the physical sequelae that it may render, which are even more serious in the elderly population. Our work identified the increased frequency of physical incapacity in elderly, especially in MB patients, which is in agreement with other findings in the Brazilian population (38–40). Similarly, the greater frequency of males among MB patients has also been documented previously due to the progressive increase in MB leprosy with age (38, 39). It is known from experimental models of intracellular pathogens such as *Leishmania* (41), *Mycobacterium tuberculosis* (42), and *Paracoccidioides brasiliensis* (43) that testosterone is capable of increasing the levels of Th2 anti-inflammatory cytokines, such as IL-10 and IL-4, while estrogen induces a Th1 response, such as IL-2 and IFN-γ. These findings may explain the higher susceptibility of men for developing the multibacillary form of leprosy.

Herein, the observation of increased memory CD8^+^ cells in skin lesions of elderly patients corroborates preceding evidence that demonstrated increased subpopulations of these cells during aging (9, 44). Although T lymphocytes are essential for the elimination of *M. leprae*, many subpopulations have reduced activation and proliferation due to the absence of the CD28 costimulatory molecule. CD8^+^CD28^−^ T cells exhibit a senescence profile along with severe reduction in telomere function due to a long replication history (45). Although other cells may express CD8 molecules, such as NK (CD8+CD3–CD56+) cells (46), it is highly likely that these CD8+ cells found in the skin of our patients studied herein are T lymphocytes. NK cells are also present in granulomatous diseases, such as tuberculosis and tuberculoid leprosy (47). Nevertheless, few studies disclosed the frequency of these cells in skin lesions of patients affected by such diseases.

While reduced expression of CD28 in peripheral blood T lymphocytes of MB patients has been previously described (24, 25), the current work provides new information correlating these data with age. We observed lower expression of CD28 molecules in CD8^+^ of elderly PB patients in comparison with young patients with the same clinical form. These findings could compromise the activation and proliferation of these cells, favoring bacilli proliferation in elderly patients. Furthermore, CD8^+^CD28^−^ cells disclosed its ability to suppress immune activity, which could hamper the activation and proliferation of Th lymphocytes through different mechanisms, such as TGF-β and IL-10 secretion and expression of PDL-1 and FasL ligands (20, 21). Nonetheless, CD8^+^CD28^−^ T lymphocytes may also reduce the capacity of dendritic cells and monocytes to present *M. leprae*-derived antigens, through increased expression of *Ig-like transcript 3* (ILT3) and *4* (ILT4) (48). In summary, increased expression of senescent leukocytes in elderly patients could constitute a permissive environment that favors *M. leprae* replication and spread.

Our data are even more striking in the elderly PB patient group since they not only present lower CD8^+^CD28^+^ cells in skin lesions but showed increased detection of anti-CMV IgG antibodies in serum. Longitudinal studies have demonstrated that elderly people have a reduced capacity to resist new infections due to an excess of memory leukocytes, especially CD8^+^CD28^−^. Another contributing factor is the scarcity of naïve T lymphocytes that present a T lymphocyte receptor (TCR) repertoire (49–51). Studies suggest that T cell subsets frequencies may be caused, in part, by latent CMV control (52, 53). In fact, CMV seropositivity seems to be a determinant factor of life expectancy, CMV^+^ individuals who present other risk factors, such as increase in CD8^+^CD28^−^ cells tend to have a shorter life span (54, 55).

As age advances, a decline of distinct intensity in adaptive immune functions is observed. It is speculated that this decline may be explained by regulatory T lymphocytes (Treg), however, these data are still inconsistent. Some studies describe a reduction in Treg (CD4^+^CD25^+^) suppressive activity during aging (54), while others show that blood Treg frequency is higher in the elderly and that their depletion improves conventional CD4^+^ T lymphocyte activity in these individuals (55). Here, expression of two receptors (PD-1 and LAG3) related to the suppression mechanisms of Treg cells does not appear to be associated to age in leprosy patients. Our results show that, regardless of age, MB patients present higher expression levels of these two receptors in skin samples than PB patients. These results are in accordance with the fact that MB patients present a permissive environment for the proliferation of *M. leprae*, considering their high bacilli counts in cutaneous lesions.

LAG3 receptor function seems to be influenced by different cytokines as well as by persistent antigen exposure (56). We found evidence that corroborates other studies showing that high bacillary load in MB patients may provide an environment that increases immunosuppressive action of Treg through LAG3. This could hinder the containment of bacilli, since LAG3 tends to increase Treg activity in an environment with high antigen exposure (57, 58). Similarly, increased PD-1 expression hampers effector T lymphocyte actions in containing *M. leprae* proliferation, resulting in high bacillary load, a characteristic of MB leprosy (59). For example, in a chronic infection model, blocking of PD-1 interaction with its ligands with nivolumab was able to revert the exhaustion of T lymphocytes (60).

Taken together, our data suggest that changes in cell subpopulations associated with aging may facilitate the appearance of signs and symptoms of leprosy in elderly people exposed to *M. leprae*. Thus, the accumulation of memory CD8^+^ and reduced frequency of CD8^+^CD28^+^ cells in elderly patients constitute important immunosenescence changes that compromise the activation of resistance mechanisms against *M. leprae*. Future studies are necessary to evaluate the senescent cells response to bacterial challenge, confirm their hyporesponsiveness when compared to non-senescent cells and still, verify the functional profile of these subsets. On this basis, the data shown in this study provide information and perspectives on the implications of immunosenescence in leprosy progression, which can be extended to other models of chronic infectious diseases.

## Data Availability Statement

The original contributions presented in the study are included in the article/Supplementary Material, further inquiries can be directed to the corresponding author/s.

## Ethics Statement

The studies involving human participants were reviewed and approved by Institutional Ethics Committee of Oswaldo Cruz Foundation/FIOCRUZ (permit protocol number 27052919.0.0000.5248). The patients/participants provided their written informed consent to participate in this study.

## Author Contributions

DE: conceptualization. ES, MMo, RL, and DE: funding acquisition. PS, KC, TL-C, MMe, and JL: performed the experiments. JN, RL, and ES: clinical follow-up. PS, TL-C, MMe, FL, MMo, and DE: analyzed the data and writing. PS, MMo, and DE: review and editing. All authors contributed to the article and approved the submitted version.

## Conflict of Interest

The authors declare that the research was conducted in the absence of any commercial or financial relationships that could be construed as a potential conflict of interest.

## Supplementary Material

Supplementary Figure 1 PD-1 ligands gene expression in blood and skin lesion samples. Expression of *PDCD1LG1* gene on **(A)** whole blood samples and **(B)** skin samples, and *PDCD1LG2* gene on **(C)** whole blood samples and **(D)** skin specimens. The results of gene expression of these PD-1 ligands are represented in normalized expression values. In the whole blood, the analysis was performed by Bio mark's microfluidic-based qPCR technology and in the cutaneous fragments, analysis was performed by RT-qPCR. Each circle represents an individual, and horizontal bars indicate the mean. Kruskal-Wallis test with Dunn's multiple comparison test correction was used to compare the groups.

Supplementary Table 1 Oligonucleotides used in the study.

## References

[1] Scollard DM Adams LB Gillis TP Krahenbuhl JL Truman RW Williams DL. The continuing challenges of leprosy. Clin Microbiol Rev. (2006) 19:338–81. 10.1128/CMR.19.2.338-381.2006 16614253 PMC1471987

[2] Foss NT Motta AC. Leprosy, a neglected disease that causes a wide variety of clinical conditions in tropical countries. Mem Inst Oswaldo Cruz. (2012) 107(Suppl.1):28–33. 10.1590/S0074-02762012000900006 23283450

[3] Scollard DM Truman RW Ebenezer GJ. Mechanisms of nerve injury in leprosy. Clin Dermatol. (2015) 33:46–54. 10.1016/j.clindermatol.2014.07.008 25432810

[4] Lockwood DN Saunderson PR. Nerve damage in leprosy: a continuing challenge to scientists, clinicians and service providers. Int Health. (2012) 4:77–85. 10.1016/j.inhe.2011.09.006 24029146

[5] Silva SR Illarramendi X Tempone AJ Silva PH Nery JA Monteiro AM. Downregulation of PHEX in multibacillary leprosy patients: observational cross-sectional study. J Transl Med. (2015) 13:296. 10.1186/s12967-015-0651-5 26362198 PMC4566286

[6] Britton WJ Lockwood DN. Leprosy. Lancet. (2004) 363:1209–19. 10.1016/S0140-6736(04)15952-7 15081655

[7] Ridley DS Jopling WH. Classification of leprosy according to immunity. A five-group system. Int J Lepr Other Mycobact Dis. (1966) 34:255–73. 5950347

[8] World Health Organization (WHO). Guide to Eliminate Leprosy as a Public Health Problem. 1st ed. Geneva: World Health Organization (1995).

[9] Boyd SD Liu Y Wang C Martin V Dunn-Walters DK. Human lymphocyte repertoires in ageing. Curr Opin Immunol. (2013) 25:511–5. 10.1016/j.coi.2013.07.007 23992996 PMC4811628

[10] Fülöp T Larbi A Pawelec G. Human T cell aging and the impact of persistent viral infections. Front Immunol. (2013) 4:271. 10.3389/fimmu.2013.00271 24062739 PMC3772506

[11] Goronzy JJ Fang F Cavanagh MM Qi Q Weyand CM. Naïve T cell maintenance and function in human aging. J Immunol. (2015) 194:4073–80. 10.4049/jimmunol.1500046 25888703 PMC4452284

[12] Carvalho JC Araújo MG Coelho-Dos-Reis JGA Peruhype-Magalhães V Alvares CC Moreira ML. Phenotypic and functional features of innate and adaptive immunity as putative biomarkers for clinical status and leprosy reactions. Microb Pathog. (2018) 125:230–9. 10.1016/j.micpath.2018.09.011 30195647

[13] Silva PHL Santos LN Mendes MA Nery JAC Sarno EN Esquenazi D. Involvement of TNF-Producing CD8+ effector memory T cells with immunopathogenesis of erythema nodosum leprosum in leprosy patients. Am J Trop Med Hyg. (2019) 100:377–85. 10.4269/ajtmh.18-0517 30652669 PMC6367624

[14] Vallejo AN Brandes JC Weyand CM Goronzy JJ. Modulation of CD28 expression: distinct regulatory pathways during activation and replicative senescence. J Immunol. (1999) 162:6572–9. 10352273

[15] Esensten JH Helou YA Chopra G Weiss A Bluestone JA. CD28 costimulation: from mechanism to therapy. Immunity. (2016) 44:973–88. 10.1016/j.immuni.2016.04.020 27192564 PMC4932896

[16] Bour-Jordan H Esensten JH Martinez-Llordella M Penaranda C Stumpf M Bluestone JA. Intrinsic and extrinsic control of peripheral T-cell tolerance by costimulatory molecules of the CD28/ B7 family. Immunol Rev. (2011) 241:180–205. 10.1111/j.1600-065X.2011.01011.x 21488898 PMC3077803

[17] Huff WX Kwon JH Henriquez M Fetcko K Dey M. The evolving role of CD8+CD28- immunosenescent T cells in cancer immunology. Int J Mol Sci. (2019) 20:2810. 10.3390/ijms20112810 31181772 PMC6600236

[18] Pera A Caserta S Albanese F Blowers P Morrow G Terrazzini N. CD28null pro-atherogenic CD4 T-cells explain the link between CMV infection and an increased risk of cardiovascular death. Theranostics. (2018) 8:4509–4519. 10.7150/thno.27428 30214635 PMC6134924

[19] Teteloshvili N Dekkema G Boots AM Heeringa P Jellema P de Jong D. Involvement of MicroRNAs in the aging-related decline of CD28 expression by human T cells. Front Immunol. (2018) 9:1400. 10.3389/fimmu.2018.01400 29967621 PMC6015875

[20] Liu Z Tugulea S Cortesini R Suciu-Foca N. Specific suppression of T helper alloreactivity by allo-MHC class I-restricted CD8+CD28- T cells. Int Immunol. (1998) 10:775–83. 10.1093/intimm/10.6.775 9678758

[21] Chen X Liu Q Xiang AP. CD8+CD28- T cells: not only age-related cells but a subset of regulatory T cells. Cell Mol Immunol. (2018) 15:734–6. 10.1038/cmi.2017.153 29375130 PMC6141529

[22] Liu Q Zheng H Chen X Peng Y Huang W Li X. Human mesenchymal stromal cells enhance the immunomodulatory function of CD8(+)CD28(-) regulatory T cells. Cell Mol Immunol. (2015) 12:708–18. 10.1038/cmi.2014.118 25482073 PMC4716622

[23] Reiser J Banerjee A. Effector, memory, and dysfunctional CD8(+) T cell fates in the antitumor immune response. J Immunol Res. (2016) 2016:8941260. 10.1155/2016/8941260 27314056 PMC4893440

[24] Sridevi K Neena K Chitralekha KT Arif AK Tomar D Rao DN. Expression of costimulatory molecules (CD80, CD86, CD28, CD152), accessory molecules (TCR alphabeta, TCR gammadelta) and T cell lineage molecules (CD4+, CD8+) in PBMC of leprosy patients using *Mycobacterium leprae* antigen (MLCWA) with murabutide and T cell peptide of T rat protein. Int Immunopharmacol. (2004) 4:1–4. 10.1016/j.intimp.2003.09.001 14975355

[25] Palermo Mde L Trindade MÂ Duarte AJ Cacere CR Benard G. Differential expression of the costimulatory molecules CD86, CD28, CD152 and PD-1 correlates with the host-parasite outcome in leprosy. Mem Inst Oswaldo Cruz. (2012) 107(Suppl.1):167–73. 10.1590/S0074-02762012000900024 23283468

[26] Dagur PK Sharma B Kumar G Khan NA Katoch VM Sengupta U. Mycobacterial antigen(s) induce anergy by altering TCR- and TCR/CD28-induced signalling events: insights into T-cell unresponsiveness in leprosy. Mol Immunol. (2010) 47:943–52. 10.1016/j.molimm.2009.11.009 20018378

[27] Guerreiro LTA Robottom-Ferreira AB Ribeiro-Alves M Toledo-Pinto TG Rosa Brito T Rosa PS. Gene expression profiling specifies chemokine, mitochondrial and lipid metabolism signatures in leprosy. PLoS ONE. (2013) 8:e64748. 10.1371/journal.pone.0064748 23798993 PMC3683049

[28] Ramakers C Ruijter JM Lekanne Deprez RH Moorman AFM. Assumption-free analysis of quantitative real-time PCR data. Neurosci Lett. (2003) 339:62–6. 10.1016/S0304-3940(02)01423-4 12618301

[29] Ruijter JM Ramakers C Hoogaars W Bakker O van den Hoff MJB Karlen Y. Amplification efficiency: linking baseline and bias in the analysis of quantitative PCR data. Nucleic Acids Res. (2009) 37:e45. 10.1093/nar/gkp045 19237396 PMC2665230

[30] Rutledge R Stewart D. A kinetic-based sigmoidal model for the polymerase chain reaction and its application to high-capacity absolute quantitative real-time PCR. BMC Biotechnology. (2008) 8:47. 10.1186/1472-6750-8-47 18466619 PMC2397388

[31] R Core Team. R: A Language and Environment for Statistical Computing. (2020). Available online at: http://cran.r-project.org/ (accessed July 2, 2020).

[32] Pfaffl MW. A new mathematical model for relative quantification in real-time RT-PCR. Nucleic Acids Res. (2001) 29:e45. 10.1093/nar/29.9.e45 11328886 PMC55695

[33] Vandesompele J De Preter K Pattyn F Poppe B Van Roy N De Paepe A. Accurate normalization of real-time quantitative RT-PCR data by geometric averaging of multiple internal control genes. Genome Biol. (2002) 3:34. 10.1186/gb-2002-3-7-research0034 12184808 PMC126239

[34] Perkins JR Dawes JM Orengo C McMahon SB Bennett DL Kohl M. ReadqPCR and NormqPCR: R packages for the reading, quality checking and normalisation of RT-qPCR quantification cycle (Cq) data. BMC Genomics. (2012) 13:296. 10.1186/1471-2164-13-296 22748112 PMC3443438

[35] McElhaney JE Effros RB. Immunosenescence: what does it mean to health outcomes in older adults? Curr Opin Immunol. (2009) 4:418–24. 10.1016/j.coi.2009.05.023 19570667 PMC2725188

[36] Hakim FT Gress RE. Immunosenescence: deficits in adaptive immunity in the elderly. Tissue Antigens. (2007) 70:179–89. 10.1111/j.1399-0039.2007.00891.x 17661905

[37] Cunha LL Perazzio SF Azzi J Cravedi P Riella LV. Remodeling of the immune response with aging: immunosenescence and its potential impact on COVID-19 immune response. Front Immunol. (2020) 11:1748. 10.3389/fimmu.2020.01748 32849623 PMC7427491

[38] Oliveira JSS Reis ALMD Margalho LP Lopes GL Silva ARD Moraes NS. Leprosy in elderly people and the profile of a retrospective cohort in an endemic region of the Brazilian Amazon. PLoS Negl Trop Dis. (2019) 13:e0007709. 10.1371/journal.pntd.0007709 31479442 PMC6743788

[39] Nobre ML Illarramendi X Dupnik KM Hacker MA Nery JA Jerônimo SM. Multibacillary leprosy by population groups in Brazil: Lessons from an observational study. PLoS Negl Trop Dis. (2017) 11:e0005364. 10.1371/journal.pntd.0005364 28192426 PMC5325588

[40] Matos TS Carmo RFD Santos FGB Souza CDF. Leprosy in the elderly population and the occurrence of physical disabilities: is there cause for concern? Ann Bras Dermatol. (2019) 94:243–5. 10.1590/abd1806-4841.20198067 31090837 PMC6486081

[41] Snider H Lezama-Davila C Alexander J Satoskar AR. Sex hormones and modulation of immunity against leishmaniasis. Neuroimmunomodulation. (2009) 16:106–13. 10.1159/000180265 19212130 PMC2760305

[42] Bini EI Mata Espinosa D Marquina Castillo B Barrios Payán J Colucci D Cruz AF. The influence of sex steroid hormones in the immunopathology of experimental pulmonary tuberculosis. PLoS ONE. (2014) 9:e93831. 10.1371/journal.pone.0093831 24722144 PMC3983091

[43] Pinzan CF Ruas LP Casabona-Fortunato AS Carvalho FC Roque-Barreira MC. Immunological basis for the gender differences in murine *Paracoccidioides brasiliensis* infection. PLoS ONE. (2010) 5:e10757. 10.1371/journal.pone.0010757 20505765 PMC2873977

[44] Gupta S Bi R Su K Yel L Chiplunkar S Gollapudi S. Characterization of naive, memory and effector CD8+ T cells: effect of age. Exp Gerontol. (2004) 4:545–50. 10.1016/j.exger.2003.08.013 15050289

[45] Effros RB Boucher N Porter V Zhu X Spaulding C Walford RL. Decline in CD28+ T cells in centenarians and in long-term T cell cultures: a possible cause for both in vivo and *in vitro* immunosenescence. Exp Gerontol. (1994) 29:601–9. 10.1016/0531-5565(94)90073-6 9435913

[46] McKinney EF Cuthbertson I Harris KM Smilek DE Connor C Manferrari G. A CD8+ NK cell transcriptomic signature associated with clinical outcome in relapsing remitting multiple sclerosis. Nat Commun. (2021) 12:635. 10.1038/s41467-020-20594-2 33504809 PMC7840761

[47] Sadhu S Mitra DK. Emerging concepts of adaptive immunity in leprosy. Front Immunol. (2018) 9:604. 10.3389/fimmu.2018.00604 29686668 PMC5900054

[48] Vlad G Cortesini R Suciu-Foca N. License to heal: bidirectional interaction of antigen-specific regulatory T cells and tolerogenic APC. J Immunol. (2005) 174:5907–14. 10.4049/jimmunol.174.10.5907 15879080

[49] Reker-Hadrup S Strindhall J Kollgaard T Seremet T Johansson B Pawelec G. Longitudinal studies of clonally expanded CD8 T cells reveal a repertoire shrinkage predicting mortality and increased number of dysfunctional cytomegalovirus-specific T cells in the elderly. J Immunol. (2006) 176:2645–53. 10.4049/jimmunol.176.4.2645 16456027

[50] Wikby A Månsson IA Johansson B Strindhall J Nilsson SE. The immune risk profile is associated with age and gender: findings from three Swedish population studies of individuals 20-100 years of age. Biogerontology. (2008) 9:299–308. 10.1007/s10522-008-9138-6 18369735

[51] Derhovanessian E Larbi A Pawelec G. Biomarkers of human immunosenescence: impact of Cytomegalovirus infection. Curr Opin Immunol. (2009) 21:440–5. 10.1016/j.coi.2009.05.012 19535233

[52] Schwanninger A Weinberger B Weiskopf D Herndler-Brandstetter D Reitinger S Gassner C. Age-related appearance of a CMV-specific high-avidity CD8+ T cell clonotype which does not occur in young adults. Immun Ageing. (2008) 5:14. 10.1186/1742-4933-5-14 19014475 PMC2596076

[53] Nikolich-Zugich J. Ageing and life-long maintenance of T-cell subsets in the face of latent persistent infections. Nat Rev Immunol. (2008) 8:512–22. 10.1038/nri2318 18469829 PMC5573867

[54] Tsaknaridis L Spencer L Culbertson N Hicks K LaTocha D Chou YK. Functional assay for human CD4+CD25+ Treg cells reveals an age-dependent loss of suppressive activity. J Neurosci Res. (2003) 74:296–308. 10.1002/jnr.10766 14515359

[55] Lages CS Suffia I Velilla PA Huang B Warshaw G Hildeman DA. Functional regulatory T cells accumulate in aged hosts and promote chronic infectious disease reactivation. J Immunol. (2008) 181:1835–48. 10.4049/jimmunol.181.3.1835 18641321 PMC2587319

[56] Graydon CG Balasko AL Fowke KR. Roles, function and relevance of LAG3 in HIV infection. PLoS Pathog. (2019) 15:e1007429. 10.1371/journal.ppat.1007429 30653605 PMC6336228

[57] Do JS Visperas A Sanogo YO Bechtel JJ Dvorina N Kim S. An IL-27/Lag3 axis enhances Foxp3+ regulatory T cell-suppressive function and therapeutic efficacy. Mucosal Immunol. (2016) 9:137–45. 10.1038/mi.2015.45 26013006 PMC4662649

[58] Huang CT Workman CJ Flies D Pan X Marson AL Zhou G. Role of LAG-3 in regulatory T cells. Immunity. (2004) 21:503–13. 10.1016/j.immuni.2004.08.010 15485628

[59] Chaves AT Ribeiro-Junior AF Lyon S Medeiros NI Cassirer-Costa F Paula KS. Regulatory T cells: Friends or foe in human *Mycobacterium leprae* infection? Immunobiology. (2018) 223:397–404. 10.1016/j.imbio.2017.11.002 29150026

[60] Nguyen LT Ohashi PS. Clinical blockade of PD1 and LAG3-potential mechanisms of action. Nat Rev Immunol. (2015) 15:45–56. 10.1038/nri3790 25534622

