# Supplementary figures and images for: Presence of Senescent and Memory CD8+ Leukocytes as Immunocenescence Markers in Skin Lesions of Elderly Leprosy Patients
Source: Front Immunol. 2021 Mar 11;12:647385. doi: 10.3389/fimmu.2021.647385 (PMC7991105; doi:10.3389/fimmu.2021.647385)

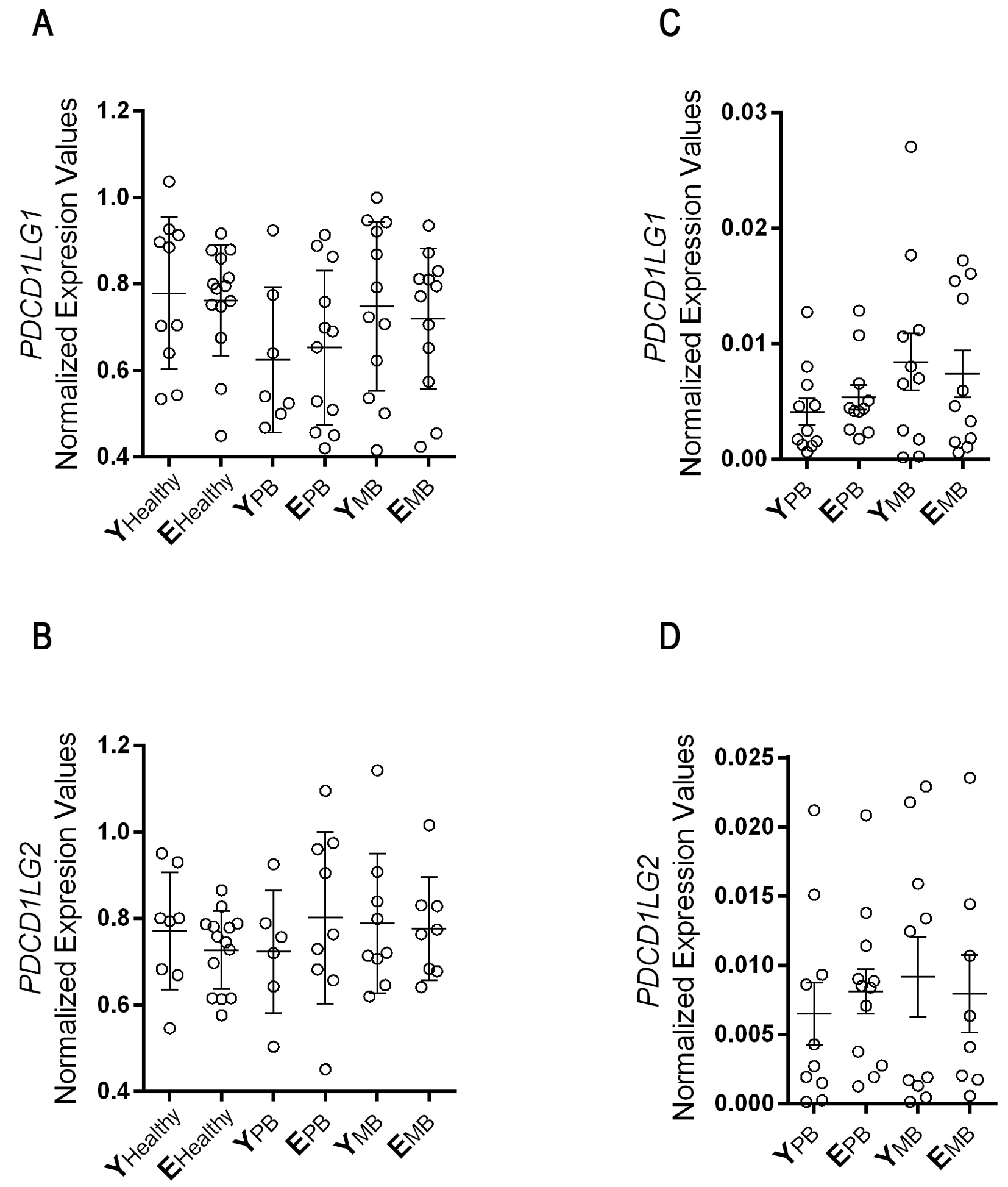

Supplement: Supplementary Figure 1 — PD-1 ligands gene expression in blood and skin lesion samples. Expression of PDCD1LG1 gene on (A) whole blood samples and (B) skin samples, and PDCD1LG2 gene on (C) whole blood samples and (D) skin specimens. The results of gene expression of these PD-1 ligands are represented in normalized expression values. In the whole blood, the analysis was performed by Bio mark's microfluidic-based qPCR technology and in the cutaneous fragments, analysis was performed by RT-qPCR. Each circle represents an individual, and horizontal bars indicate the mean. Kruskal-Wallis test with Dunn's multiple comparison test correction was used to compare the groups. [file Image_1.TIF]
