# Supplementary material for: Presence of Senescent and Memory CD8+ Leukocytes as Immunocenescence Markers in Skin Lesions of Elderly Leprosy Patients
Source: Front Immunol. 2021 Mar 11;12:647385. doi: 10.3389/fimmu.2021.647385 (PMC7991105; doi:10.3389/fimmu.2021.647385)
Supplement: Supplementary Table 1 — Oligonucleotides used in the study. [file Table_1.DOCX]

**Table S1: Oligonucleotides used in the study.**

| Gene | Official full name | Sequence 5' 🡪 3' | Gene bank |
| --- | --- | --- | --- |
| *PDCD1* | Programmed cell death 1 | Fwd - GCACGAGGGACAATAGGAGC | NM_005018.3 |
|  |  | Rev - ATAGTCCACAGAGAACACAGGC |  |
| *PDCD1LG1* | Programmed cell death 1 ligand 1 | Fwd - AGGGAGAATGATGGATGTGAA | NM_014143.4 |
|  |  | Rev - CGTCTCCTCCAAATGTGTATCA |  |
| *PDCD1LG2* | Programmed cell death 1 ligand 2 | Fwd - TTCACCAGATAGCAGCTTTATTCA | NM_025239.4 |
|  |  | Rev - GCTCCAAGGTTCACATGACTTC |  |
| *LAG3* | Lymphocyte activating 3 | Fwd - CACCTCCTGCTGTTTCTCATC | NM_002286.6 |
|  |  | Rev - GTCTTGGTCGCCACTGTCTT |  |
| *CD28* | CD28 molecule | Fwd - TTAGCTGCAAGTATTCCTACAATCTC | NM_006139.4 |
|  |  | Rev - AGCACTATCCAGTCCTTTGTGAAG |  |
| *RPL13* | Ribosomal protein L13 | Fwd - GACAAGAAAAAGCGGATGGT | NM_000977.4 |
|  |  | Rev - GTACTTCCAGCCAACCTCGT |  |
| *RPS16* | Ribosomal protein S16 | Fwd - GCGCACGCTACAGTACAAG | NM_001020.6 |
|  |  | Rev - AGATGGACTGACGGATAGCATA |  |
| *RPL35* | Ribosomal protein L35 | Fwd - CGAGTCGTCCGGAAATCCAT | NM_007209.4 |
|  |  | Rev - GGCTTGTACTTCTTGCCCTTG |  |

***Fwd****, forward primer;* ***Rev****, reverse primer.*
